# Echocardiography in the Ventilated Patient: What the Clinician Has to Know

**DOI:** 10.3390/jcm14010077

**Published:** 2024-12-27

**Authors:** Fiorella Chiara Delle Femine, Diego D’Arienzo, Biagio Liccardo, Maria Concetta Pastore, Federica Ilardi, Giulia Elena Mandoli, Simona Sperlongano, Alessandro Malagoli, Matteo Lisi, Giovanni Benfari, Vincenzo Russo, Matteo Cameli, Antonello D’Andrea

**Affiliations:** 1 Cardiology Unit, Department of Medical Translational Sciences, Monaldi Hospital, University of Campania “Luigi Vanvitelli”, 80136 Naples, Italy; fiorellachiara.df@gmail.com (F.C.D.F.); diegodarienzo12@gmail.com (D.D.); sperlongano.simona@gmail.com (S.S.); v.p.russo@libero.it (V.R.); 2 Department of Cardiology, Monaldi Hospital, University of Campania “Luigi Vanvitelli”, 80136 Naples, Italy; liccardob@gmail.com; 3 Department of Medical Biotechnologies, Division of Cardiology, University of Siena, 53100 Siena, Italy; pastore2411@gmail.com (M.C.P.); giulia_elena@hotmail.it (G.E.M.); matteo.cameli@yahoo.com (M.C.); 4 Department of Advanced Biomedical Sciences, Division of Cardiology, Federico II University Hospital, Via S. Pansini 5, 80131 Naples, Italy; federica.ilardi@unina.it; 5 Division of Cardiology, Nephro-Cardiovascular Department, Baggiovara Hospital, 41126 Modena, Italy; ale.malagoli@gmail.com; 6 Department of Cardiovascular Disease—AUSL Romagna, Division of Cardiology, Ospedale S. Maria Delle Croci, Viale Randi 5, 48121 Ravenna, Italy; matteo.lisi@hotmail.it; 7 Section of Cardiology, Department of Medicine, University of Verona, 37129 Verona, Italy; giovanni.benfari@gmail.com; 8 Department of Cardiology, Umberto I Hospital, 84014 Nocera Inferiore, Italy

**Keywords:** mechanical ventilation, echocardiography, venous return, TAPSE, inferior vena cava, stroke volume, right ventricle, fluid challenge, diaphragm

## Abstract

Heart and lung sharing the same anatomical space are influenced by each other. Spontaneous breathing induces dynamic changes in intrathoracic pressure, impacting cardiac function, particularly the right ventricle. In intensive care units (ICU), mechanical ventilation (MV) and therefore positive end-expiratory pressure (PEEP) are often applied, and this inevitably influences cardiac function. In ventilated patients, the use of positive pressures leads to an increase in intrathoracic pressure and, consequently, to a reduction in the right ventricular preload and thus cardiac output. The clinician working in the intensive care unit must be able to assess the effects MV has on the heart in order to set it up appropriately and to manage any complications. The echocardiographic evaluation of the ventilated patient has the main purpose of studying the right ventricle; in fact, they are the ones most affected by PEEP. It is therefore necessary to assess the size, thickness, and systolic function of the right ventricle. In the mechanically ventilated patient, it may be difficult to assess the volemic status and fluid responsiveness, in fact, the study of the inferior vena cava (IVC) is not always reliable in these patients. In patients with MV, it is preferable to assess fluid responsiveness with dynamic methods such as the end-expiration occlusion (EEO) test, passive leg raise (PLR), and fluid challenge (FC). The study of the diaphragm is also essential to identify possible complications, manage weaning, and provide important prognostic information. This review describes the basis for echocardiographic evaluation of the mechanically ventilated patient with the aim of supporting the clinician in managing the consequences of MV for heart–lung interaction.

## 1. Introduction

The heart is in the thorax cavity where it occupies the juxtacardiac fossa and is, therefore, a pressure chamber within a pressure chamber. For this reason, the heart and the lungs are inevitably influenced by each other [1]. Some examples are chronic pulmonary heart in patients with chronic obstructive pulmonary disease or pulmonary hypertension. The heart and the lungs work as a single functional unit whose purpose is to ensure an adequate body oxygen supply. The first to study heart–lungs mechanical interaction was the physiologist Stephen Hales. He demonstrated that, by inserting a glass column into the carotid artery of a horse, blood pressure varied with breathing. Nowadays, the clinician cannot fail to be conscious of the influence that the respiratory system has on the cardiovascular system; in fact, this interaction plays a fundamental role, especially in critical patients requiring mechanical ventilation (MV). In these patients, hemodynamics, already compromised by the underlying pathology, can be further destabilized by incorrect ventilation to the point of even fatal outcomes. Regardless of what type of mechanical ventilation the patient requires, from fully controlled to simple continuous positive airway pressure (CPAP), the common denominator is the use of positive pressures.

### Statistical Methodology

This review was conducted in accordance with PRISMA guidelines [2].

## 2. Heart–Lung Interaction in Spontaneous Ventilation

**Figure 1:**
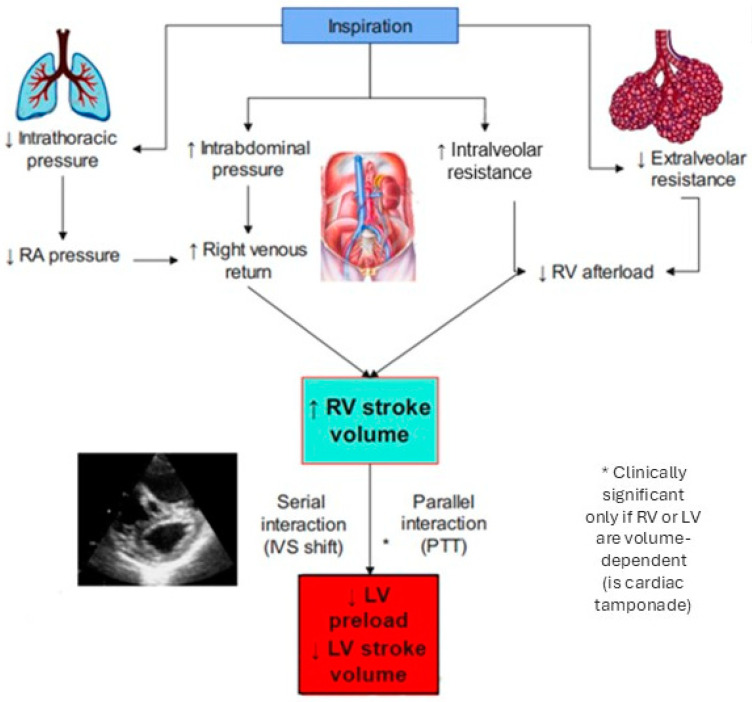
During spontaneous inspiration, there is a decrease in intrathoracic pressure (and thus right atrial pressure) and an increase in intra-abdominal pressure. This combined effect leads to increased right ventricular preload. Concurrently, during inspiration, there is an overall reduction in pulmonary vascular resistances (prevalence of reduction in extralveolar vascular resistances exceed the increase in intra-alveolar vascular resistances) resulting in a net reduction in right ventricular afterload. Increased venous return and reduced right afterload results in increased right ventricular stroke volume (RVSV). By serial and parallel interaction (see text), this reduces the left ventricular afterload. Only when the left ventricle is preload dependent (e.g., severe hypovolemia, cardiac tamponade, etc.) is there a reduction in the left ventricular stroke volume (LVSV).

Heart and lungs work together to ensure sufficient oxygen supply to the body. In fact, the oxygen delivery (DO2) is the product of the oxygen content in arterial blood for cardiac output [3]. Cardiac hemodynamics can be influenced by two pressures: intrathoracic pressure and transpulmonary pressure (the difference between alveolar pressure and the intrathoracic pressure). During inspiration, intrathoracic pressure becomes more negative, and it is transmitted to the right atrium, whereas the lowering of the diaphragm causes an increase in intrabdominal pressure; this causes an increase in the gradient between the right atrium and the extra thoracic veins, therefore favoring venous return. Thus, during spontaneous inspiration, there is an increase in the preload and consequently the right ventricular (RV) stroke volume. The pulmonary vascular bed is directly affected by intrapleural pressure; even during spontaneous ventilation it does not determine clinically significant alterations [4]. During inspiration, the increment of lung volume compresses the alveolar vessels, resulting in an increase in the right ventricular afterload, but the effect of the enhanced venous return predominates and consequently the right stroke volume increases [5]. The relation between pulmonary vascular resistance (PVR) and lung volume is bimodal with minimal resistance near the functional residual capacity (FRC), which is the volume remaining in the lungs after a normal, passive exhalation. There are two types of intraparenchymal pulmonary vessels: intra-alveolar vessels that are compressed by increasing lung volume and extra-alveolar vessels that expand as lung volume increases. At volumes below the FRC, effects on extra-alveolar vessels prevail and PVR decreases. Above the FRC, effects on intra-alveolar vessels prevail and PVR increases [6,7]. Also, hypoxia leads to vasoconstriction and therefore to an increase in the pulmonary vascular resistance and right ventricular afterload [8]. The left and right ventricles are affected by two types of interaction that explain the ventricular interdependence: parallel interaction and serial interaction; it should be specified that the following interaction and physiological variations are negligible in healthy subjects, but they assume great relevance during mechanical ventilation and cardiovascular pathology like pulmonary hypertension. The first is explained by the increase in right ventricular tele-diastolic volume; this causes a shift in the interventricular septum to the left with a subsequent reduction in left ventricular filling and, consequently, to cardiac output [9]. The latter is caused by the increment in the right ventricular stroke volume during inspiration, not immediately transmitted to an increase in the left ventricular preload because, even in healthy patients, the pulmonary transit time (PTT, the time needed for blood to be transferred from the right ventricle to the left ventricle) is almost 8–10 s [10]. So, during the next expiration, the left ventricle preload is increased. These two phenomena are the explanations for the reduction in stroke volume during inspiration. Relative to the left ventricle (LV), during inspiration there is a reduction in stroke volume. This is due to ventricular interdependence (shown above) and the fact that the LV has to fight both the negative intrapleural pressure in order to contract, and to overcome an increased afterload. This increased afterload results from peripheral vessels, which are not within the thorax, being unaffected by intrapleural pressure. In summary, during the various phases of the respiratory cycle, the stroke volume emitted by the two ventricles varies in a complementary manner (during inspiration the stroke volume of the right ventricle increases and that of the left ventricle decreases, and vice versa during exhalation), so that the average stroke volume during some cardiac cycles (average of tens of seconds) is equivalent for the two ventricles [1](Figure 1).

## 3. Heart–Lung Interaction in Mechanical Ventilation

**Figure 2:**
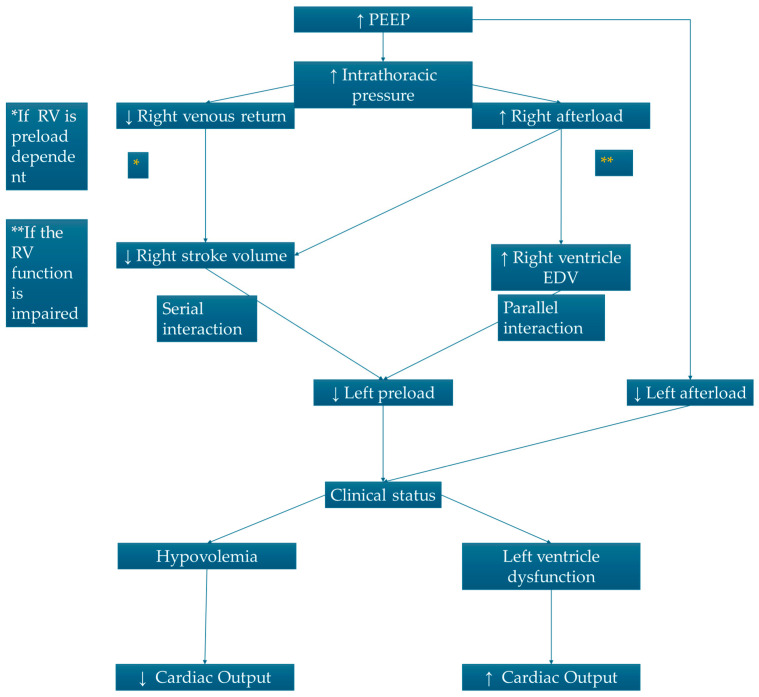
Hemodynamic changes in positive pressure ventilation.

In ventilated patients, the use of positive pressures leads to an increase in intrapleural pressure and consequently a reduction in the transmural gradient of the right atrium, which results in a reduction in venous return, right ventricular stroke volume, and, consequently, to cardiac output. This reduction in the right ventricular preload can be advantageous in some patients such as those in acute pulmonary edema but can be dangerous in patients where the preload is already reduced, such as in septic and/or hypovolemic shock [11]. Mechanical ventilation should be set up with extreme caution using low positive pressures (i.e., max. 8 mL/kg tidal volume) in patients in which right filling is compromised as in cardiac tamponade, or in those where the right ventricular afterload is already increased as in pulmonary hypertension or pulmonary embolism. The use of positive end-expiratory pressure (PEEP) plays two contrasting roles on vascular pulmonary resistances and consequently on the right ventricle afterload: on the one hand, it helps to ventilate more alveoli and consequently reduces vasoconstriction due to hypoxia reducing the right ventricle afterload. On the other hand, it increases intra-alveolar vascular resistance by increasing lung volumes. Thus, the use of high volumes leads to an increase in the right ventricular afterload, which in combination with a reduction in venous return results in a reduced right ventricle stroke volume, and so a reduction in the left ventricular preload (serial ventricular interdependence, see above) and, consequently, to cardiac output. The influence of mechanical ventilation on cardiac output has always been a matter of controversy. On one hand, the cardiac output suffers from reduced filling due to reduced venous return, but on the other hand, the increase in the intrathoracic pressure (and so an increase on the pericardial pressure) leads to a reduction in left ventricular transmural pressure (difference between intraventricular pressure and intrapericardial pressure), reducing intramural tension and thus the left ventricle stroke work. In addition, the increase in intrathoracic pressure generates a gradient in intravascular pressures between the thoracic aorta (on which PEEP acts by increasing transmural pressure) and the abdominal aorta, which is exposed to neutral atmospheric pressure, thus determining a “pump effect” between the thoracic and abdominal aortas, in effect reducing the afterload to the left ventricle [7]. Which of the major left ventricle effects (preload reduction and afterload reduction) potentially may affect cardiac output more depends on the patient’s specific clinical condition. Indeed, patients with congestive heart failure (CHF) and left ventricular dysfunction may benefit from both preload and afterload reduction, as this can improve the coupling between reduced ventricular function and reduced afterload, leading to increased cardiac output [12,13]. On the other hand, in hypovolemic patients with preserved contractile function, the effect on the preload reduction will predominate, adding to the reduced mean systemic filling pressure (MSFP) due to the reduced “stressed volume” of venous vessels [1] (Table 1, Figure 2).

## 4. Echocardiographic Evaluation in Mechanical Ventilation

**Figure 3:**
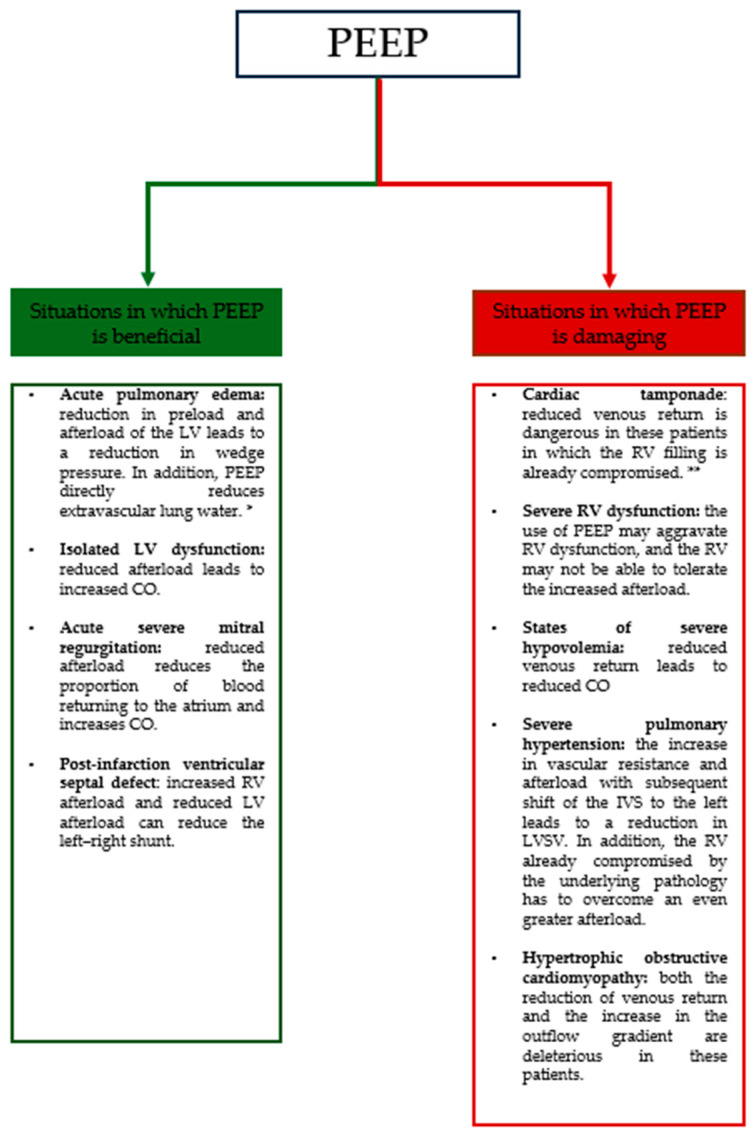
Conditions in which PEEP is useful. CO: cardiac output, IVS: interventricular septum, LV: left ventricular, LVSV: left ventricular stroke volume, PEEP: positive end-expiratory pressure, RV: right ventricular. * Kuhn et al. Management of Mechanical Ventilation in Decompensated Heart Failure [15]. ** Madhivathanan et al. Perioperative implications of pericardial effusions and cardiac tamponade [16].

The clinician who must manage mechanically ventilated patients has to be able to manage not only the patient from a respiratory aspect, but above all, to monitor the influence of ventilation on hemodynamics to prevent and/or control any complications that may occur and to choose the ventilatory support best suited to patient’s condition (Figure 3). Echocardiography is a valuable tool for rapid, noninvasive bedside assessment of right ventricular (RV) function in MV patients, which can provide important prognostic information. In a study by Hongmin Zhang et al. [14], septic patients requiring mechanical ventilation were divided into four groups based on RV function:

- Normal RV function;
- Right ventricular failure (RVF) without right ventricular systolic dysfunction (RVSD);
- RVSD without RVF;
- Combined RVF and RVSD.

RVF was defined as a ratio of RV to LV end-diastolic area (RVEDA/LVEDA) ≥ 0.6 combined with a central venous pressure (CVP) ≥ 8 mmHg. RVSD was defined as a tricuspid annular plane systolic excursion (TAPSE) < 16 mm or a fractional area change (FAC) < 35%. Patients with combined RVF and RVSD had the highest levels of positive end-expiratory pressure (PEEP) and the highest 30-day mortality rate [14].

## 5. Right Ventricular Echocardiographic Evaluation

Dimensions: Right ventricle dilation can occur during MV. The right ventricle dimensions are obtained in end-diastole from the four-chamber view from the apical window; it is very important to obtain a well-oriented image; by changing the cutting planes, this may appear larger or smaller than it really is. To obtain an optimal window, the transducer should be rotated to the maximum plane obtainable and positioned above the cardiac apex with the plane passing through the center of the left ventricle cavity [17]. RV dilatation is defined by a diameter >41 mm at the base and 35 mm at the medium level. The basal diameter of the right ventricle should also be compared with the diameter of the left ventricle. Indeed, in patients with pulmonary embolism, an RV/LV ≥ 0.9 was found to be an independent predictor of hospital mortality.

RV wall thickness: It should be measured from the subcostal window, in end-diastole, using the zoom at a distance from the tricuspid ring equal to the distance of the tricuspid anterior flap when it is fully open and parallel to the wall. Care should be taken to exclude the pericardium, trabeculae, and papillary muscles from the measurement. A value greater than 5 mm is indicative of hypertrophy [17,18]. In patients undergoing mechanical ventilation, especially when this is performed for prolonged periods, there may be an increase in the right ventricle wall thickness due to increased afterload [19].

**Figure 4:**
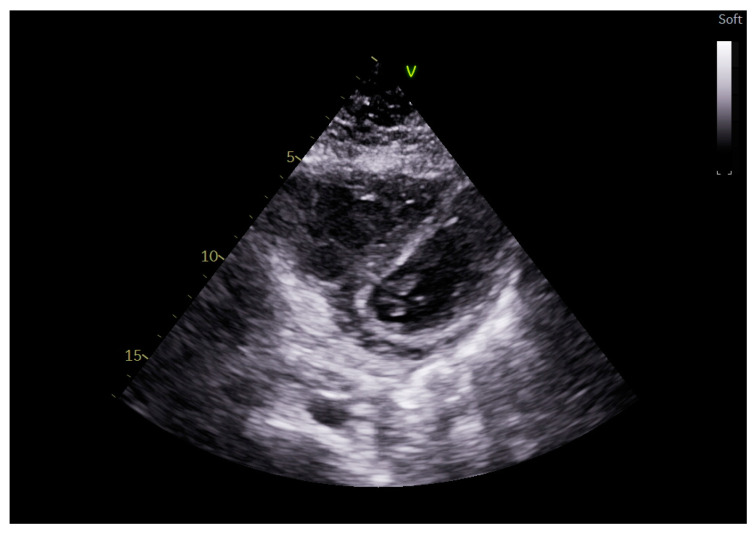
D-shaped appearance of the left ventricle.

Interventricular septum: During mechanical ventilation, there can be a posterior displacement of the interventricular septum due to both the increase in intrathoracic pressure per se and the potential increase in right afterload when using excessive PEEP or tidal volume (TV). Generally, this paradoxical septal movement affects the entire cardiac cycle, whereas the movement due to volume overload is only diastolic (as in large interatrial defects). This phenomenon can reduce cardiac output due to the principle of ventricular interdependence [20]. This movement can create in the parasternal short axis a D-shaped appearance of the left ventricle. Thus, there will be an increase in the eccentricity index represented by the ratio of the anteroposterior to the septolateral diameter with a cutoff of 1 [17]. It is not always easy to visually identify reverse movement of the septum in the short-axis view. For less obvious forms, a more objective method such as left ventricular radial strain could be employed. Indeed, it has been shown that in patients ventilated for moderate–severe ARDS by COVID-19 and with paradoxical septal movement, there was an alteration in the radial strain of the left ventricle. However, further research is needed to elucidate this association [21] (Figure 4).

Fractional area change (FAC): The percentage of FAC is calculated as (end-diastolic area–end-systolic area)/end-diastolic area × 100. It is reduced if less than 35%. The FAC can be measured in four chambers, including in the image the entire right ventricle in both systole and diastole. The RV area at end-systole and end-diastole should be plotted, taking care to include the trabeculae in the RV cavity [17,18].

Tricuspid annular plane systolic excursion (TAPSE) and S’ wave: The TAPSE is an index of RV longitudinal function. It is widely used for its simplicity of measurement and reproducibility. It is measured in four apical chambers by placing the M-mode on the lateral tricuspid annulus and measuring the excursion; the cutoff is 17 mm. Although it has high prognostic value, it represents the function of a single RV segment. It may be reduced in cases of normal right-sided function such as, for example, in cardiac surgery or be normal in regional hypokinesia [22]. Another index of RV longitudinal function is the S’ wave; it is measured laterally at the level of the tricuspid annulus by PW–Doppler in systole, and correct alignment is important for correct sampling. It is normal when greater than or equal to 10 cm/s [23].

Right ventricular strain: Right ventricular longitudinal strain (RVLS) is the application of speckle-tracking echocardiography (STE) for the study of the longitudinal function of the right ventricle, making it independent of preload [24]. It can be determined through an image obtained from an RV-focused apical four-chamber (A4C) view, synchronized with the ECG. Optimizing the frame rate between 60 and 90 frames per second, as close to the heart rate as possible, is crucial for accurate measurement. There is no single, universally accepted approach to RVLS analysis. Some authors consider the free wall RVLS (fwRVLS), which analyzes only three segments of the right ventricle free wall (basal, middle, and apical). The normal value for fwRVLS is less than −23%, with less negative values indicating RV dysfunction. Other authors advocate for a six-segment approach, including the three segments of the posterior interventricular septum (IVS). The normal value in this case is less than −20% [25]. Performing the STE for the right ventricle can be challenging in critically ill patients, as the directly retrosternal position of the right ventricle, poor patient compliance, and presence of physical barriers (dressings, electrodes, etc.) can make acquisition of RV-focused A4C views more complex. However, Vos et al. demonstrated that suitable images for RVLS analysis could be obtained in about 70% of patients; moreover, they observed that patients with reduced RVLS also had higher acute physiology, chronic health evaluation (APACHE) IV score, and 30-day mortality, even in a small group of patients who had regular TAPSE and S’-TDI values. Another noteworthy finding was that RVLS increased (less negative) in mechanically ventilated patients as PEEP increased [26]. Shuyuan Wang et al. found reduced RVGLS values in mechanically ventilated patients with acute respiratory failure compared to those who were not mechanically ventilated. Notably, this reduction in RVGLS was independent of PEEP levels. RVGLS demonstrated superior sensitivity in detecting right ventricular dysfunction compared to traditional parameters like TAPSE and FAC [27]. McErlane et al. analyzed fwRVGLS in mechanically ventilated patients with COVID-19, and found that those with fwRVGLS > 20% had a higher 30-day mortality [28].

Systolic pulmonary arterial pressure (sPAP): The pressure difference between the atrium and the right ventricle can be determined with the transthoracic echocardiogram by means of the tricuspid regurgitant peak velocity (TRV). TRV can be obtained by positioning the continuous Doppler at the level of the tricuspid regurgitant. Using the simplified Bernoulli equation, we can estimate the sPAP, which is given by 4× TRV2 + right atrial pressure (RAP) [29]. RAP is estimated based on the size and collapsibility of the inferior vena cava (IVC). The most recent guidelines recommend using only TRV to avoid errors due to inaccurate RAP estimation. This has even more plausibility considering that IVC size and collapsibility are not always reliable in ventilated patients [30]. The TRV is not accurate in cases of pulmonary stenosis and severe tricuspid insufficiency.

Atrial dimensions and function: Current understanding of the prognostic value of atrial assessment in mechanically ventilated patients remains limited. However, it is known that PEEP influences atrial pressure and function. Left atrial volume can be measured using the biplane method of disks by tracing the endocardial borders in four and two chambers. Right atrial volume, on the other hand, can be measured by using either the single plane area–length or the disks summation technique in the apical four chambers [18]. Studies have shown a reduction in both left and right atrial volumes in MV patients as PEEP values increase. As with atrial volume, it has been shown that both the right and left atrial strain decreases with increasing PEEP in MV patients [31]. Left atrial strain is measured by tracing the endocardial border in the four-chamber view, while right atrial strain is assessed in the apical view by focusing on the right ventricle [32]. Strain measurements offer the advantage of being independent of the angle of insonation, and of being a semi-automatic technology. This is a significant advantage in critical care settings where obtaining optimal echocardiographic views can be challenging.

Estimation of left ventricular filling pressures: As we mentioned previously, the use of PEEP leads to a decrease in the left ventricular preload and afterload. This can be beneficial in patients with an elevated pulmonary capillary wedge as they can help the left ventricle with a sort of unloading. More attention should be given to patients with reduced left preload, such as in severe hypovolemia or severe right ventricular dysfunction [33]. Echocardiographic evaluation of left atrial pressure (LAP), which correlates most closely with wedge pressure, is therefore useful. It is then necessary to assess the transmitral flow pattern with the E/A ratio. Other fundamental parameters are septal e‘ velocity (altered if less than 7), lateral e’ velocity (altered if less than 10), E/e’ ratio (altered if greater than 14), left atrial volume (altered if greater than 34 mL/m^2^), and tricuspid jet velocity (altered if greater than 2.8 m/s), In patients with a preserved ejection fraction, the presence of at least three of these positive parameters is indicative of diastolic dysfunction and therefore elevated left atrial pressure (LAP). In patients with reduced ejection fraction, an E/A ratio ≥ 2 is indicative of elevated LAP, while an E/A ratio ≤ 0.8 and E ≤ 50 cm/s is indicative of normal LAP. In intermediate cases, other parameters should be used [34]. While echocardiographic assessment of LAP can be useful in selecting patients who may benefit from mechanical ventilation, it can be misleading in patients who are already mechanically ventilated. In these patients, increased pericardial pressure can lead to a reduction in left ventricular end-diastolic volume and consequently an increase in end-diastolic pressure, which, however, is not indicative of preload in this case [35]. In these cases, invasive hemodynamic parameters will be necessary to determine preload.

Inferior vena cava (IVC): In the intensive care unit, there is often a need to quickly assess a patient’s volemic status to understand whether the patient needs fluids. One of the quickest methods is the assessment of the inferior vena cava and its variability with breath phases (ΔIVC). It should be assessed in longitudinal scanning from the subcostal window 1 to 2 cm from the junction with the right atrium. Normal values are a diameter ≤ 21 mm collapsing more than 50%. Under spontaneous breathing conditions, the IVC collapses in inhalation due to increased venous return. In ventilated patients, assessment of the inferior vena cava is unreliable, although there are several studies with conflicting results. During MV with positive pressures, there will be an increase in intrapleural pressure at the end of exhalation, which will lead to a reduction in the pressure gradient between the right atrium and systemic veins, resulting in an increase in the diameter of the IVC at the end of exhalation and a reduction in ΔIVC. According to some authors, there is differing validity of ΔIVC depending on the ventilation settings; it is a good predictor of fluid response in patients ventilated with TV ≥ 8 mL/kg and PEEP ≤ 5 cm H_2_O, poor if TV < 8 mL/kg or PEEP > 5 cm H_2_O [36].

Internal jugular vein: There are various ways to study the internal jugular vein (IJV); the most validated in ventilated patients is the distensibility index, calculated as (IJVmax − IJVmin)/IJVmin, expressed as a percentage. It is sampled at the level of the cricoid cartilage, in the transverse plane by measuring the maximum and minimum diameters with the M-mode [37]. A IJV distensibility index > 18% showed good sensitivity and specificity for predicting fluid responsiveness [38] (Table 2).

## 6. Echocardiography and Volume Management

**Figure 5:**
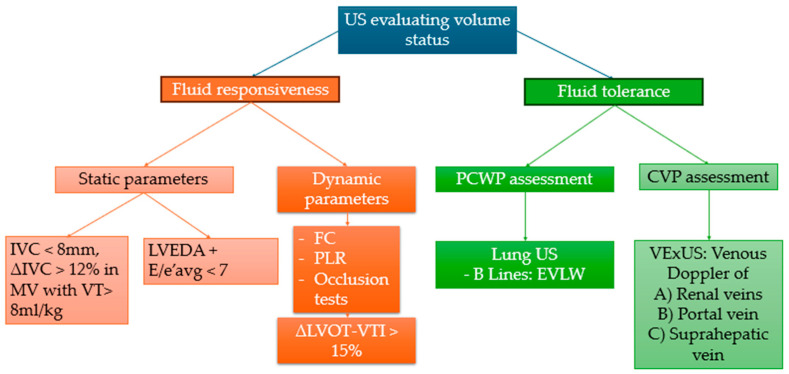
Ultrasound in assessing the volemic status of ventilated patients involves the analysis of fluid responsiveness and fluid tolerance. CVP: central vein pressure, EVLW: extravascular lung water, FC: fluid challenge, LVEDA: left ventricular end-diastolic area, MV: mechanical ventilation, IVC inferior vena cava, ΔIVC: (IVC max diameter—IVC min diameter)/IVC max diameter, LVOT-VTI: left ventricular outflow tract velocity time integral; PCWP: pulmonary capillary wedge pressure, PLR: passive leg raise, US: ultrasound, VExUS: venous excess ultrasound, VT: tidal volume.

In critically ill patients, particularly those with left ventricular dysfunction, it is difficult to manage the volemic state, as there is a frequent fear that water overload may lead the patient into pulmonary edema [39]. Despite this concern, the initial therapeutic approach in a patient in the context of a low output syndrome is volemic optimization, followed possibly by the introduction of vasoactive drugs or the use of mechanical circulatory support systems [40]. The use of bedside ultrasound can be a noninvasive and inexpensive tool that allows multiple observations to predict and monitor over time the patient’s therapeutic response. Echocardiographic examination in the critical area, including in ventilated patients, in order to assess the efficacy or possible harm caused by fluid administration, is based on the evaluation of data to define fluid responsiveness and fluid tolerance (Figure 5).

Fluid responsiveness:

The classical definition of fluid responsiveness is the increase in SV of 15% after administration of a bolus of 500 mL of crystalloids in 10–15 min [41]. Echocardiographic methods to reveal a patient’s fluid responsiveness are distinguished into static and dynamic. As respiratory dynamics affect the right ventricular preload, static methods appear more fallacious and are influenced by numerous variables, so greater reliability is attributed to dynamic methods. Conceptually, patients with greater changes in SV during inspiration will be more likely fluid responders [41].

Static parameters include:

- IVC diameter in ventilated patient predicts fluid responsiveness with a specificity of 95% when the end-expiratory diameter is <8 mm [41], while a reduction in IVC diameter during inspiration of 18% could predict fluid responsiveness in septic, ventilated patients with 90% of sensibility and specificity [42]. Commonly, in patients with right heart failure and severe tricuspid regurgitation, it is common to observe IVC dilatation regardless of volume status.
- Left ventricular end diastolic area (LVEDA), as measured with transesophageal echocardiography in ICU at an angle of 0° or 90° (respectively four chamber view and two chamber view), is not sufficient alone to predict fluid responsiveness. It is worth mentioning that one observational study found that the combination of LVEDA (<21 cm^2^) and the ratio of PW Doppler of left ventricular early filling (E peak wave) and the tissue Doppler of the mitral anulus (e’avg) as E/e’avg (<7) could predict fluid responsiveness in patients undergoing coronary surgeries without heart failure with reduced left ventricular function (HFrEF) and with an AUC of 0.9 [43]. Static parameters are less useful in patients with severe left ventricular dysfunction as they are unable to evaluate the response of the cardiovascular system to the fluid administration. Therefore, the data derived from these parameters must be considered only as part of the clinical–instrumental evaluation of the patient, and necessarily integrated with the dynamic parameters, especially in ventilated and critically ill patients with ventricular dysfunction.

Dynamic parameters:

Echocardiographic dynamic parameters are based on measuring changes in the left ventricular stroke volume (SV) after a preload-enhancing maneuver. One of the quickest and most effective methods to assess the change in SV following a diagnostic maneuver (listed below) is to observe the change in the velocity–time integral (VTI) obtained by pulsed wave Doppler (PW), placing the sample volume at the left ventricular outflow tract (LVOT) in an apical five-chamber image (A5C) [44].

(1) Fluid challenge (FC): The fluid challenge is based on the administration of a 500 mL bolus of crystalloids in 10–15 min and subsequent observation of SV change. A change greater than 15% defines fluid responsiveness [45]. When monitoring SV with pulse-contour analysis, a minifluid challenge can be useful in place of echocardiography. By administering 100 cc of crystalloids in one minute, the 6% change in SV (which turns out to be too small to be detected by the echocardiographic method of VTI on LVOT) predicts fluid responsiveness to a global bolus of 250 cc of crystalloids [46]. In a small observational study of 30 patients admitted to cardio-thoracic intensive care, it was observed that in patients responsive to a fluid challenge (FC), but not in nonresponders, administration of a bolus of crystalloids during FC improved ventriculo-arterial coupling, reducing arterial elastance and consequently systemic arterial resistances [47].
(2) Passive leg raise (PLR): PLR works analogous to the FC; however, instead of administering a bolus of fluid from the outside, elevation of the legs and supination of the trunk results in a shift of approximately 300–500 cc of blood from the splanchnic venous compartment and lower extremities to the right ventricle; thus, similar to the FC, the cutoff for defining fluid responsiveness is a SV change of 15% (calculated as the change in LVOT-VTI). It is essential to remember that for proper performance of the test, the patient must initially be positioned in a semisupine position, with the trunk tilted about 45°, and then, concurrently with leg raising, the trunk is moved to a supine position. In addition, it is necessary to exclude the influence of adrenergic stimulation determined by pain [48].
(3) Occlusion tests: In ventilated patients, as described above, inspiration augments PEEP, which reduces venous return to the right ventricle. Thus, if the left ventricle is preload dependent, through serial and parallel interaction, then the left ventricle SV is reduced while the opposite happens during end-expiration. This concept is the basis for the end-expiration occlusion (EEO) test and end-inspiration occlusion (EIO) test. Ventilation interruption during end-expiration for 15 s (EEO) followed by ventilation interruption during end-inspiration for 15 s (EIO) can enhance the observation of LVSV variation in echocardiography. The combination of EEO and EIO was tested in 30 ventilated patients and the absolute variation in SV of 13% was able to predict fluid responsiveness with a sensitivity and specificity of 93% (AUC 0.973) [49]. In general, dynamic parameters are useful to predict fluid responsiveness in ventilated patients with tidal volume (TV > 8 mL/kg), while in particular, the EEO test, PLR, and FC are useful to assess even patients with lung protective ventilation (TV < 8 mL/kg) [50].

Choosing the method to evaluate fluid responsiveness depends on the specific clinical context with which the physician must interact. For example, patients who have arterial or large femoral vascular access (e.g., short-term mechanical circulation support systems) will be unable to practice PLR. Echocardiography-assessed FC presents the natural limit represented by the patient’s acoustic window, particularly valid in patients who have undergone recent cardiac surgery in which it is not always easy to obtain adequate alignment of the ultrasound beam to the LVOT. Occlusion tests can be performed only in patients who are not breathing spontaneously.

Fluid tolerance cannot be defined through a quantitative variable; it represents the ability of the patient’s cardio-respiratory system to tolerate a fluid bolus without progressing to organ dysfunction [51]. While hemodynamic parameters (CVP and PCWP) can help define poor fluid tolerance (e.g., if CVP > 12 mmHg and PCWP > 18 mmHg), these parameters are not always readily available and depend on the interaction with biventricular function. An easy way to assess fluid tolerance is through bedside ultrasonography, specifically to assess pulmonary congestion status with lung ultrasound (LUS), which indirectly detects left ventricular dysfunction (systolic and/or diastolic) and splanchnic congestion status through Doppler profile assessment of renal, suprahepatic, and portal veins (venous excess ultrasound, VExUS) defining right ventricular dysfunction (systolic and/or diastolic) [52]. The presence of extravascular lung water (EVLW) evidenced at LUS by the presence of B-lines (>3–4 per field of observation) in the ultrasonographic fields predicted by the Blue protocol defines impaired fluid tolerance; in addition, the occurrence following a FC of more than two B-lines per field of observation predicts nonresponsiveness to fluids with an AUC of 0.74 [53,54]. Through the integrated analysis of portal venous flows, renal venous flows, and suprahepatic venous flows, it is possible to obtain the VExUS score, which dichotomizes patients into fluid tolerant (VExUS score 0–1) and fluid intolerant (VExUS score 2–3) [55] (Figure 5). 

## 7. Diaphragm Anatomy and Physiology

The diaphragm is a dome-shaped muscle when in the relaxed phase. It separates the thoracic from the abdominal cavity. It consists of two structures: the central aponeurosis and a muscular portion. The central aponeurosis consists of noncontractile tissue of a fibrous nature, and it is shaped like a clover with an anterior and two lateral leaves. The muscular portion, on the other hand, is composed of fast and slow muscle fibers and is divided into three parts: the sternal, the costal, and the lumbar. It attaches internally to the aponeurosis and externally to the ribs, to the sternum, and to the lumbar spine [56]. The portion of the diaphragm that inserts into the chest wall is called the apposition zone. During inhalation, the contraction of the muscle fibers and their consequent shortening leads to the lowering of the dome, allowing the expansion of the thoracic cavity; it also favors the closure of the gastro-esophageal junction to prevent gastro-esophageal reflux during inhalation. In relation to the pressures, during inhalation, there is a reduction in intrapleural pressure and subsequent entry of air, and oppositely an increase in intra-abdominal pressure. The difference between the intra-abdominal pressure and the intrapleural pressure constitutes the transdiaphragmatic pressure, which represents the actual force produced by the contraction of the diaphragm [57].

## 8. Diaphragmatic Dysfunction in Mechanically Ventilated Patients in the Intensive Care Unit

Alterations in diaphragmatic function are not uncommon in intensive care; these can be up to 80% in patients who require mechanical ventilation for a prolonged period and have difficulty weaning [58,59]. One of the disorders that can be observed in critically ill patients in intensive care is diaphragmatic paralysis. Unilateral diaphragmatic paralysis may occur in patients who have been subject to coronary artery bypass surgery. It can occur either from a direct nerve injury due to its close anatomical proximity to the internal mammary artery, or from degeneration induced by low temperatures due to ice for cardiac cooling. Phrenic nerve injury has shown impact on short- and medium-term prognosis in bypass and chronic obstructive pulmonary disease (COPD) patients [59]. The first to report on ventilation-induced diaphragm dysfunction (VIDD) was Levine in 2008 [60]. In the first twenty-four hours of using mechanical ventilation, diaphragm muscle weakness can already be observed in some individuals. This may be caused by an incorrect setting of mechanical ventilation. Ewan C Goligher et al. identified four mechanisms underlying diaphragmatic myotrauma in mechanically ventilated patients: it can be caused by over-assistance of the ventilator and thus atrophy of the muscles, under-assistance of the ventilator and thus damage due to muscle fatigue, but also by dyssynchrony, or the use of a high value of PEEP [61]. The development of mechanical ventilation-induced diaphragmatic muscle weakness can be a cause of difficult weaning from ventilation and is correlated with a longer need for ventilatory assistance and a prolonged stay in intensive care [62]. Diaphragmatic dysfunction secondary to mechanical ventilation has been demonstrated in numerous studies on both animals and humans [60,63,64]. At the molecular level, increased oxidative stress, activation of proteolytic pathways, and mitochondrial dysfunction with loss of muscle strength have been shown to underlie this process [65]. Very often MV acts on a diaphragm already compromised by other causes such as sepsis-related myopathy [66]. In addition, the use of certain drugs such as volatile anesthetics, propofol, or corticosteroids represents a risk factor in the development of VIDD [67].

## 9. Echocardiographic Assessment of Diaphragmatic Function

**Figure 6:**
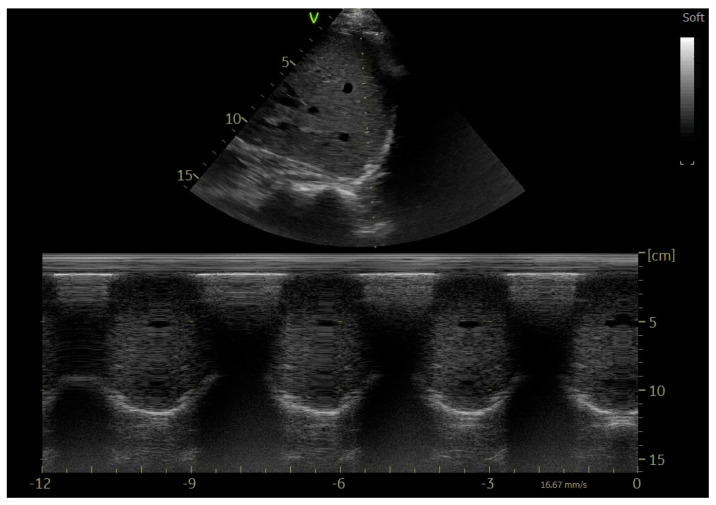
Diaphragm movement in M-mode.

Considering the necessity for the clinician working in the intensive care unit to be able to recognize diaphragmatic dysfunction, the easiest usable, low-cost, radiation-free tool available at the patient’s bedside is ultrasound. This technique finds its main purpose in the assessment of diaphragmatic function through the study of movement, quantification of the thickness, and thickening fraction. Diaphragmatic thickness can be measured in M-mode using a high-frequency probe (≥10 Mhz). The probe should be positioned on the mid-axillary line, approximately between the eighth and eleventh rib; in this position the apposition zone can be seen. This position is preferred because it is the least affected by cranio-caudal variability in the thickness of the diaphragm itself [68]. The right flank is preferable so that the liver acts as ultrasound window. On ultrasound, the diaphragm appears as a structure consisting of three echogenic layers: two outer hyperechogenic layers represented by the pleural and peritoneal membranes, and internally by the hypoechogenic muscular layer. Measurements should be taken at the end of exhalation, excluding the outer layers. Diaphragmatic thickness differs widely depending on where it is measured; for this reason it is advisable to mark a reference point and always take measurements at the same point. Other variables that can influence diaphragmatic thickness are gender, a sedentary lifestyle [69,70], and position [71]. Reference values are 0.19 ± 0.04 cm in men and 0.14 ± 0.03 cm in women. If the diaphragmatic thickness is measured during maximal inspiratory and expiratory effort, the thickening fraction can be obtained [tdi% = (tdi end inspiration − tdi end expiration)/tdi endexpiration × 100]; this value is preferable to basic thickness as it is less affected by interindividual variability. Reference values range from 60% to 260% for the left hemidiaphragm and from 57% to 200% for the right hemidiaphragm in a seated patient [72]. Another fundamental parameter to assess is diaphragmatic movement. It can be obtained by using a low-frequency probe (1–5 MHz), placing the probe just below the costal arch, on the right hemiclavicular line, and pointing it dorsally. This will use the liver as an acoustic window. Actually, the probe can also be positioned to the left, in this case using the spleen as the acoustic window. After displaying the diaphragm movement in B-mode (qualitative mode), the clinician can switch to M-mode, where the respiratory movement made by the diaphragm will appear as a hyperechogenic curve (Figure 6). The slope of this curve will then be measured and expressed in cm/s [62]. The difference between the position of the diaphragm at functional residual capacity and total lung capacity (TLC) is the maximum diaphragmatic excursion, whereas the difference between the FRC position and the end-inhalation position during resting breathing is the tidal excursion [72]. Impaired diaphragmatic movement is indicative of diaphragm muscle weakness. It is necessary not only to check that the diaphragm is moving, but also to study the relationship of the movements with the phases of breathing; in fact, in diaphragmatic paralysis there is a paradoxical movement with a rise during inhalation. Therefore, even with the use of B-mode, it is possible to rule out diaphragmatic paralysis.

## 10. Evaluation of the Diaphragm in Ventilated Patient

In ventilated patient, the use of echocardiographic monitoring of diaphragmatic function can be useful at all the steps in mechanical ventilation, as it can help to discern which patient needs mechanical ventilation [73], to diagnose possible complications, and to guide the clinician in weaning. Not all ultrasound parameters are usable in these patients; e.g., in assisted ventilation, the diaphragmatic excursion is not reliable as it may be the result of passive insufflation [74]. Goligher et al. [61] in their study showed that a reduction in diaphragmatic thickness correlated with a lower daily probability of release from ventilation, prolonged ICU admission, and a higher risk of complications. An increase in diaphragmatic thickness is associated with longer ventilation. This may be due to excessive or reduced respiratory effort. It also showed that a thickening fraction between 15% and 30% during the first three days after admission to the ICU was found to be associated with the shortest duration of ventilation [62,67]. One of the most difficult issues in intubated patients is to choose the right timing for extubation; indeed, a failed extubation prolongs the time spent in the ICU and consequently has a high prognostic impact. Three studies evaluated tdi% as an index of successful extubation, showing that this parameter has good sensitivity and specificity, with a cutoff ranging from 30 to 36% depending on the study [75,76,77].

## Figures and Tables

**Table 1:** Hemodynamic changes in spontaneous breathing versus positive pressure ventilation.

	Spontaneous Inhalation	Positive Pressure Ventilation
**Intrapleural pressure**	**↓**	**↑**
**Venous return**	**↑**	**↓**
**Right ventricular stroke volume**	**↑**	**↓**
**Pulmonary vascular resistances**	**↑**	↑↓ *
**Left ventricular stroke volume**	**↓**	**↓**
**Left ventricular afterload**	**↑**	**↓**

* The effect depends on volume: at high volumes, increased vascular resistance predominates; at low volumes, reduced vasoconstriction due to hypoxia relief is dominant.

**Table 2:** Echocardiographic parameters to evaluate right ventricular response to mechanical ventilation.

Parameter	Evaluating	Pathologic Cutoff	How to	Pros	Cons	Tips
**RV diameters**	RV dimension and relationship with LV	RVD1 (basal) > 41 mm RVD2 (medial) > 35 mm	A4C view end-diastole	Easy and rapid to obtain	Need for good acoustic window. Risk of overestimation when using a shorted image	It is useful to reduce the size of the ultrasound window to increase frames per second (FPS), sometimes it may be necessary to change the tilt of the ultrasound beam to improve the definition of the endocardial edges.
**RV wall thickness**	Index of RV hypertrophy	RVWT > 5 mm	Subcostal view, end-diastole	Easy and rapid to obtain	Subcostal view may be suboptimal in ventilated patients	Using zoom, measure at a distance from the tricuspid ring equal to the distance of the tricuspid anterior flap when it is fully open and parallel to the wall
**IVS**	Index of RV pressure or volume overload	Posterior displacement of the IVS towards the LV. Eccentricity index > 1.	SAX papillarymuscles view	No measure needed. Index of both volume and pressure overload	//	Sisto-diastolic D-shape points towards pressure overload (afterload increase) Only diastolic D-Shape points toward volume overload (es. Congenital heart disease).
**FAC**	Global RV systolic function	<35%	A4C view	Prognostic implication as indicator of RVSD	Time consuming, need for endocardial borders to been seen	It is useful to reduce the size of the ultrasound window to increase frames per second (FPS), sometimes it may be necessary to change the tilt of the ultrasound beam to improve the definition of the endocardial edges.
**TAPSE, S’**	Longitudinal RV Systolic function	TAPSE < 18 mm S’ < 10 cm/s	A4C view	Prognostic implication as indicator of RVSD, Easy to obtain	May be lower after cardiac surgery, need for good ultrasound alignment	
**sPAP**	Index of RV afterload	TRV > 3.4 m/s PAPs > 40 mmHg	A4C view, PLAX view (anterior probe tilt), SAX view	If TR is more than trivial: easy to obtain, Useful for assessment of afterload changes over time and therefore adjusting PEEP and PS therapy	May be underestimated in severe TR or RVSD, May be difficult to obtain in trivial TR	Need for good ultrasound alignment
**TAPSE/sPAP ratio**	Index of RV arterial-ventricular coupling	TAPSE/PAPs < 0.5 mm/mmHg *	See above	Prognostic implication, see above TAPSE and sPAP	Combination of pitfalls for TAPSE and PAPs.	

FAC: fractional area changes, IVS: interventricular septum, PEEP: positive end-expiratory pressure, PLAX: parasternal long axis, PS: pressure support, RV: right ventricle, RVSD: right ventricular systolic dysfunction, SAX: short axis, sPAP: systolic pulmonary arterial pressure, TR: tricuspid regurgitation, TRV: tricuspid regurgitant peak velocity. * [13].
